# Nodular Fasciitis of the Hand Over the Metacarpophalangeal Joint: A Case Report

**Published:** 2008-07-24

**Authors:** Alexander M Sailon, Guy Cappuccino, Meera Hameed, Earl J Fleegler

**Affiliations:** New Jersey Medical School, University of Medicine and Dentistry of New Jersey, Newark; Division of Plastic Surgery, Department of Surgery, New Jersey Medical School, University of Medicine and Dentistry of New Jersey, Newark; Department of Pathology, New Jersey Medical School, University of Medicine and Dentistry of New Jersey, Newark

## Abstract

**Objective:** This study describes a case of nodular fasciitis involving the hand and reviews the neoplasm's pertinent clinical, histologic, and pathologic features. **Methods:** The patient's chart, operative record, histologic specimens, and relevant literature were reviewed. **Results:** We report a case of nodular fasciitis involving the hand of a 55-year-old woman that was treated with marginal excision. **Conclusions:** Nodular fasciitis is a self-limited, benign soft tissue tumor composed of fibroblasts and myofibroblasts that typically afflicts younger patients and rarely presents in the hand. Because of its presentation, it can be easily mistaken for a malignant neoplasm. However, most cases represent a reactive and therefore a polyclonal process. Marginal excision generally provides definitive treatment.

Nodular fasciitis is a self-limited, benign lesion composed of fibroblasts and myofibroblasts. Because of its rapid growth and worrisome histologic features, it may be mistaken for a sarcoma. This lesion is typically found in the proximal upper limb, however, it may rarely present in the hand.^1^–^4^ Although nodular fasciitis is benign, it has been debated whether a reactive or a neoplastic process is responsible for the aberrant proliferation. Here, we present a case of nodular fasciitis of the hand at an uncharacteristic age and discuss its clinical and pathologic features.

## CASE REPORT

A 52-year-old, right-hand-dominant African American woman presented with a 6-month history of a slowly growing painless mass over the volar aspect of the right little finger metacarpophalangeal (MCP) joint. She denied any history of trauma, infections, foreign travel, or other soft tissue masses. She denied other neurologic symptoms, hand weakness, pains, fever, chills, unexpected weight loss, or other constitutional symptoms. Her medical history was significant for carpal tunnel syndrome, hypertension, iron deficiency anemia, and asthma.

Physical examination of the hand revealed a multinodular soft tissue mass, measuring approximately 3 × 4.3 cm, involving the volar aspect over the right little finger MCP joint area. The lesion was firm, nontender, and immobile. No erythema, tenderness, warmth, or fluctuance was present in the hand. Full active and passive flexion of the little finger MCP joint was restricted, limiting opposition as well. Range of motion in the other MCP joints was normal, along with that of all the proximal and distal interphalangeal joints. Neither epitrochlear nor axillary lymphadenopathy was palpable. Sensation was fully intact throughout both hands as determined by Semmes-Weinstein monofilaments.

**Figure 1:**
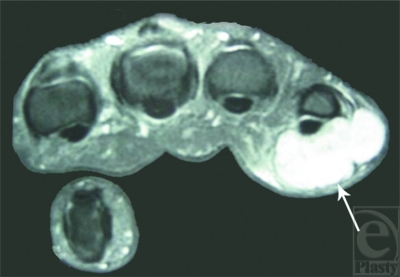
Preoperative magnetic resonance imaging (MRI). Gadolinium-enhanced, T1-weighted MRI shows a 2.3 × 2.3 × 1.3-cm lobulated, sharply circumscribed lesion (arrow) lying on the volar aspect of the little finger proximal phalanx and metacarpophalangeal joint, without cortex or tendon invasion.

Radiographs of the right hand revealed a noncalcified soft tissue mass adjacent to the ulnar aspect of the little finger proximal phalanx. Magnetic resonance imaging showed a 2.3 × 2.3 × 1.3-cm lobulated, sharply marginated lesion. The distal extent of the tumor lay directly on the volar cortex of the little finger proximal phalanx, whereas the proximal extent began at the level of the MCP joint, without invading either of them. Magnetic resonance imaging demonstrated the mass in contact with both flexor and extensor tendons, without completely encasing them (Fig 1).

**Figure 2:**
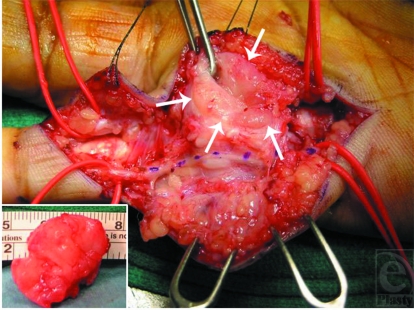
Intraoperative view. The mass (arrows) was elevated from its underlying bed. It was closely adherent to, but not invading, the little finger flexor tendons and neurovascular structures. Note the displaced digital nerve identified with methylene blue. Insert shows excised mass measuring approximately 1.5 × 3 cm.

**Figure 3:**
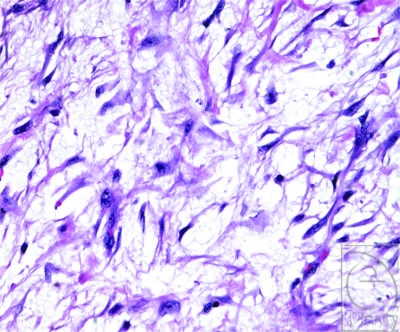
Microscopic image. The lesion consists of plump, immature-appearing fibroblasts arranged in fascicles in a myxoid and fibrous stroma (hematoxylin and eosin, ×400).

An incisional biopsy of the mass was performed through a longitudinal incision. The histologic evaluation from frozen sections revealed a predominantly fibrous lesion compatible with fibromatosis. On the basis of these results, a marginal excision of the entire mass was carried out 2 weeks after the initial procedure (Fig 2). The lesion was adherent to several structures including the neurovascular bundles of the little finger, Grayson and Cleland ligaments, the flexor tendon sheath between A-1 and A-2 pulleys, the periosteum of the proximal phalanx, and the area of the lateral band of the extensor mechanism. Adherence to these structures made dissection difficult. However, the lesion did not infiltrate these structures, so that they were all left intact. Histopathologic analysis revealed a mesenchymal lesion consisting of plump, immature-appearing fibroblasts arranged in fascicles in a myxoid and fibrous stroma (Fig 3). A zonation effect was present, with dense, hypercellular areas transitioning to areas of low cellularity with hyaline fibrosis. There were scattered inflammatory cells, extravasated red blood cells, and areas with keloid-like collagen. Mitotic figures were readily appreciated in the cellular areas, with none appearing atypical. On the basis of these findings, a final diagnosis of nodular fasciitis was made.

Postoperatively, the patient was placed in a short-arm splint and she underwent occupational therapy for range of motion and strengthening exercises. Her recovery was uneventful. Three months following surgery, she was noted to have full range of motion. She did not exhibit any signs of recurrence 12 months postoperatively.

## DISCUSSION

On the basis of the incisional biopsy, the lesion was initially thought to represent a deep fibromatosis or a desmoid tumor. Because of its tendency for slow, infiltrative growth and local recurrence, we performed a marginal excision of the mass. Histologic examination of the mass in its entirety led to the conclusive diagnosis of nodular fasciitis. Soft tissue masses in the hand may be posttraumatic, inflammatory, or neoplastic (benign vs malignant).^5^ Although generally considered a benign lesion, there has been considerable debate regarding the origins of nodular fasciitis. In particular, it remains unclear whether the lesion originates from a reactive and therefore a polyclonal process or if it represents a true neoplastic (ie, monoclonal) population.^3^ Recent cytogenetic studies have demonstrated clonal chromosomal aberrations in some cases of nodular fasciitis.^6^–^8^ However, Koizumi et al^9^ performed clonality analyses in 24 female patients with nodular fasciitis and showed a polyclonal population by a HUMARA-methylation-specific polymerase chain reaction. Therefore, most cases of nodular fasciitis represent a reactive process composed of proliferating fibroblasts and myofibroblasts. Two cases of an aggressive course resulting in bone invasion and death have been reported.^4^

Nodular fasciitis is common in young adults, aged 20–40 years. Our patient was significantly outside this age range at 52 years. In fact, only 13% of cases are found in patients older than 50.^10^ Nodular fasciitis often presents as a rapidly growing mass, 1 month or less in duration, which leads to it being mistaken for a malignant neoplasm. For this reason, a diagnosis must be aggressively and carefully sought. Nodular fasciitis is usually found as a small (<2 cm) nodular lesion, with approximately half of the patients reporting mild pain or tenderness. The lesion most commonly presents in the upper extremity, with a predilection for the volar aspect of the forearm. Lesions in the hand are extremely rare.^1^

The differential diagnosis of nodular fasciitis in the hand includes fibromatosis, fibrosarcoma, fibrous histiocytoma, Dupuytren's nodules, giant cell tumor of the tendon sheath, synovial sarcoma, and calcifying aponeurotic fibroma.^11^

After the history and physical examination (including palpation of regional lymphatics), radiographs (which are inexpensive and rapid) should be obtained. However, they generally do not provide detailed anatomic information. Magnetic resonance imaging is useful in determining tumor extent and may help establish a diagnosis. Computed tomographic scans have little utility in evaluating soft tissue masses because they often have similar attenuations as muscle.^12^

Most cases present as subcutaneous masses, but occasionally they can involve fascia and muscle. In rare instances, the mass can grow large enough to extend through the skin. Nodular fasciitis has a characteristic histologic pattern, composed predominantly of plump, immature fibroblasts in an abundance of ground substance. This imparts a loosely textured or feathery appearance. Mitotic figures are common; however, they are never atypical, unlike in sarcomas.^3^

Marginal excision generally provides definitive treatment, with recurrence being rare.^1^–^3^,^13^,^14^ During a marginal excision, the line of dissection enters the reactive zone of the tumor, an area consisting of inflammatory cells, mesenchymal cells, and neovasculature formed by the junction of the neoplasm and the surrounding normal tissue. Malignant tumors often have extensions into the reactive zone. For this reason, it is important to differentiate the lesion from a sarcoma, which may require wide margins, sacrifice of neighboring anatomic structures, or even amputation.^15^ The existence of the reactive zone makes “shelling out” these malignant masses an inadequate treatment.

In this patient, the mass was found to be strongly adherent to the surrounding structures, in contrast to other case reports that have described a mobile mass that is readily dissected.^2^,^11^ On the basis of our experience, these lesions are capable of more than simply adhering to soft tissue. The senior author (E.J.F.) previously encountered a patient where nodular fasciitis had eroded the bone near the MCP joint of the finger. Consequently, dissection can be complex and arduous.
